# Melatonin as an Oncostatic Molecule Based on Its Anti-Aromatase Role in Breast Cancer

**DOI:** 10.3390/ijms22010438

**Published:** 2021-01-04

**Authors:** Yunho Jin, Yoo Jin Choi, Kyu Heo, Seong Joon Park

**Affiliations:** Research Center, Dongnam Institute of Radiological & Medical Sciences, Busan 46033, Korea; jynh33@dirams.re.kr (Y.J.); cyj5325@dirams.re.kr (Y.J.C.); kyuh33@dirams.re.kr (K.H.)

**Keywords:** breast cancer, estrogen, aromatase, melatonin, oncostatic

## Abstract

Breast cancer is the most common type of cancer. In the developmental stages of breast cancer, estrogens are strongly involved. As estrogen synthesis is regulated by the enzyme aromatase, targeting the activity of this enzyme represents a therapeutic option. The pineal hormone melatonin may exert a suppressive role on aromatase activity, leading to reduced estrogen biosynthesis. A melatonin-mediated decrease in the expression of aromatase promoters and associated genes would provide suitable evidence of this molecule’s efficacy as an aromatase inhibitor. Furthermore, melatonin intensifies radiation-induced anti-aromatase effects and counteracts the unwanted disadvantages of chemotherapeutic agents. In this manner, this review summarizes the inhibitory role of melatonin in aromatase action, suggesting its role as a possible oncostatic molecule in breast cancer.

## 1. Introduction

Breast cancer is one of the most commonly occurring types of cancer in women worldwide, and it has been reported that estrogens are involved in regulating growth and development [1]. There are two different origins of estrogens which are the following: (1) ovarian estrogen secretion and (2) estrogen synthesis in non-ovarian tissues catalyzed by enzymes, including aromatase [2]. During the synthesis of estrogens, the enzyme aromatase behaves as a key molecule. The estrogen-synthesizing enzyme aromatase, located in estrogen-producing cells in organs such as the adrenal glands, placenta, testicles, adipose tissue, and brain, as well as the ovaries, catalyzes aromatization, which is the process that converts androgens into estrogens [2]. Since the enzyme aromatase is in charge of estrogen biosynthesis, estrogen-associated genes are known to be downregulated when this enzyme is inhibited [3]. Hence, inhibitors of this enzyme have been considered to be effective targeted therapies for breast cancer. Currently, third-generation aromatase inhibitors have been used in the treatment of estrogen-dependent breast cancers [4]. They are classified into type 1 (steroidal and noncompetitive) and type 2 (nonsteroidal and competitive) inhibitors [5]. The type 1 inhibitor exemestane irreversibly binds to the aromatase molecule, causing permanent inactivation of the enzyme [6]. In contrast, anastrozole and letrozole, the type 2 inhibitors, bind to the molecule reversibly [7]. Because the binding of estrogen to its receptor facilitates the growth of breast cancer cells, receptor-targeted cancer therapy strategies have been widely utilized [8]. Estrogen receptors are a kind of nuclear transcription factor and are engaged in numerous complex physiological actions [2]. When activated, estrogen receptors translocate into the nucleus, and then bind to DNA to regulate the various actions of diverse genes [2].

It has been suggested that adjuvant hormonal therapy is able to suppress the recurrence of hormone-dependent breast cancers [9]. Among the adjuvant breast cancer therapeutic strategies, estrogen-stimulated pathways are the most significant targets [10]. Selective estrogen receptor modulators (SERMs) and selective estrogen enzyme modulators (SEEMs) are widely used anti-estrogenic therapeutic options for hormone-dependent breast cancer. Aside from SERMs and SEEMs, aromatase inhibition may also be an attractive option for breast cancer treatment. When downregulated, the risk of breast cancer might be lowered, as aromatase is known to control estrogen biosynthesis [11]. Aromatase inhibitors appear to have higher therapeutic efficacy with less adverse effects as compared with anti-estrogen therapy [12,13,14]. While SERMs may increase the risk of endometrial cancer and thromboembolism [15], aromatase inhibitors are less likely to cause such pathological events [16].

Melatonin, the nocturnally released pineal hormone, has attracted great attention due to its pleiotropic roles. Although its major role is the regulation of the circadian rhythm, this indoleamine engages in multiple mechanisms, including neurogenesis, antioxidation, and inflammatory responses [17,18,19,20]. Apart from its versatility, this molecule has also gained a reputation as being potentially tumor suppressive, especially in hormone-dependent cancers [21]. Melatonin is able to selectively neutralize the estrogenic effects on the breast [22]. The oncostatic roles of melatonin are based on its diverse effects, including its antioxidative properties, apoptosis induction, and anticancer immunity [23]. Interestingly, melatonin is able to protect normal cells from the cytotoxicity accompanied by ionizing radiation (IR). This non-targeted effect following IR is alleviated via melatonin-mediated regulation of prostaglandins, Toll-like receptors (TLRs), and transcription factors [24]. Additionally, melatonin has been proposed to enhance the therapeutic efficacy of IR, suggesting that this hormone could be a potential radiosensitizer [25]. The radiosensitization of breast cancer cells by melatonin is mediated through reduced cell proliferation, the facilitation of cell cycle arrest, downregulated DNA repair, and increased p53 mRNA levels [22].

Intriguingly, aromatase activity in breast cancer cells is known to be regulated by melatonin [26,27]. It lowers estrogen biosynthesis by suppressing aromatase activity [28]. Multiple researchers have found receptor-targeted cancer therapies to be effective, and their transaction with melatonin would be beneficial [29,30]. In this respect, this review aimed to summarize recent findings related to the melatonin-induced inhibition of aromatase and the resultant growth suppression of hormone-dependent breast tumors.

## 2. The Role of Estrogen and Aromatase in Breast Cancer Development

### 2.1. Estrogen Promotes the Growth of Breast Cancers

Estrogens are sex hormones that control the regulation of the female reproductive system. Thanks to estrogens, the mammary gland can achieve normal development, growth, and differentiation [31]. The steroid hormone group of estrogens, which are composed of estrone (E1), estradiol (E2), and estriol (E3) is fundamental in maintaining female physiology and reproduction. Among the three main kinds of estrogens, estradiol is the most potent ligand and contributes to several of the physiological and pathophysiological mechanisms of the body’s organs [32]. Apart from their physiological and reproductive functions, estrogens have been revealed to be responsible for the growth of hormone-dependent mammary tumors [33]. Although these hormones regulate menstrual cycles and are imperative for normal reproduction, lifelong exposure to estrogens may lead to a risk of breast cancer, as evidenced by the estrogen-triggered genesis, growth, and development of hormone-dependent breast tumors [31]. Approximately 70% of breast cancers have been reported to have estrogen receptors (ERs), and estrogens are related to the risk of breast cancer [34]. It has been hypothesized that the estradiol–ERα complex might increase cell proliferation, leading to an increased risk of accumulated DNA replication errors [35]. Increased exposure to estrogens has been associated with augmented breast cancer risk [1,36]. In detail, increased estrogen exposure promotes the proliferation, invasion, metastasis, and angiogenesis of hormone-dependent breast cancers by enhancing the secretion of chemokine (C–C motif) ligand 2 (CCL2) [37]. Moreover, the fact that tamoxifen, which is the estrogen receptor antagonist approved by the Food and Drug Administration FDA, reduces breast cancer risk provides a basis for the breast cancer risk-enhancing role of estrogen [38]. Similarly, Tian et al. [39] insisted that estradiol administration increased the viability of MCF7 and cyclin G1 expression in a dose-dependent manner. According to these researchers, the cyclin G1 knockdown in MCF7 significantly limits cell viability and clonogenic ability, indicating that estradiol is related to breast cancer cell proliferation via increasing cyclin G1 expression [39]. In this context, it has been widely revealed that female hormone estrogens could represent growth signals of hormone-dependent mammary tumors.

### 2.2. Aromatase: The Major Estrogen Synthase Is Involved in Breast Cancer Development

The enzyme aromatase, which is encoded by the CYP19A1 gene, belongs to the cytochrome P450 family [40]. This enzyme complex is comprised of cytochrome P450 protein and nicotinamide adenine dinucleotide phosphate (NADPH)-cytochrome P450 reductase [41]. Since this enzyme is involved in estrogen synthesis, it is called estrogen synthase. Particularly, aromatase transforms androgens (testosterone and androstenedione) into estrogens [41]. More specifically, testosterone and androstenedione are transformed into estradiol and estrone by aromatase, respectively [42]. This aromatization of androgens includes the creation of an aromatic A ring, which is one of the distinct characteristics of estrogens, via the deletion of the C-19 methyl group [43]. The mammary adipose tissue of normal breasts has a low aromatase level via the relatively weak aromatase promoter I.4, whereas malignant cells and adjacent fibroblasts show increased aromatase expression through the activation of promoters II and I.3 [14,44,45]. These promoters are two major drivers of aromatase, accounting for more than 80% of the total aromatase expression [44,45]. Fibroblasts adjacent to mammary tumor cells cannot be differentiated into adipocytes because cytokines, such as tumor necrosis factor α (TNFα) and interleukins 6 and 11 (IL-6 and IL-11), secreted from malignant epithelial cells, suppress their differentiation, and also increase aromatase expression [14,46]. In this regard, paracrine interactions among tumor cells, fibroblasts, and endothelial cells trigger estrogen synthesis and reduce adipogenic differentiation, making mammary tumor cells prone to malignant cell growth [47,48,49]. In this way, adipose fibroblasts adjacent to mammary tumor cells increase estrogen synthesis as a response to paracrine signals from tumor cells, providing a structural and biochemical basis for the growth of breast cancer [31,50,51]. Premenopausal ovaries generate two-thirds of estrogens; other peripheral tissues create the rest [31]. While estrogens are generated from androgens in the ovaries of premenopausal women, they can no longer be synthesized from the ovaries of postmenopausal women. Instead of the non-functional ovaries, aromatase in other organs, including adipose tissue, the brain, blood vessels, skin, bone, and breast tissue, converts androgens into estrogens [14]. Particularly, adipose tissue formation in the breast of postmenopausal women could be a major source of tumor growth, as breast adipose tissue increases with age and is the main site of aromatase expression [45]. As the risk of breast cancer increases with age, postmenopausal women account for the majority of breast cancer patients [14]. Among the postmenopausal females diagnosed with breast cancer, hormone-dependent breast cancer is found in 75% of these patients, justifying the necessity of endocrine treatment [52,53]. In postmenopausal women, the peripheral conversion of androgen into estrogen is mainly mediated by aromatase. Therefore, aromatase inhibition may lead to a reduced breast cancer risk, as well as decreased estrogen levels. Indeed, aromatase inhibition has been suggested as a standard treatment for postmenopausal women with breast cancer [54]. In addition, aromatase has attracted the interest of researchers, since much higher levels of aromatase and estrogens are found in breast tumor tissue as compared with normal tissue [55]. Indeed, the levels of aromatase mRNA have been found to be higher in postmenopausal females as compared with premenopausal women [56]. Moreover, elevated leptin, high-sensitivity C-reactive protein (hsCRP), adiponectin, high-density cholesterol levels, and an increased adipocyte diameter have been found in postmenopausal females [56]. It can be inferred that an increase in aromatase levels and adipose dysfunction may lead to a rise in the incidence of hormone-dependent breast cancer in postmenopausal women. Interestingly, the pineal hormone melatonin has been reported to inhibit aromatase expression in breast cancer cells. Hence, the aromatase-suppressive role of melatonin and its consequent contribution to a reduced risk of hormone-dependent breast cancer are discussed in the following sections.

## 3. Aromatase-Inhibitory Role of Melatonin

### 3.1. Melatonin Modulates the Estrogenic Effects in Breast Cancer

Since estrogens play a role in breast cancer growth, the estrogen signaling pathway has become a main focus of cancer therapy. In order to neutralize the effects of estrogens, melatonin could be utilized as it regulates the enzymes responsible for local estrogen synthesis [28]. In human mammary tumors, aromatase, sulfatase, and 17β-HSD1, which are in charge of converting androgens into estrogens, hydrolyzing estrone sulfates to estrone, and transforming estrone to the potent 17β-estradiol, respectively, are reportedly overexpressed [48]. In contrast, estrogen sulfotransferase tends to be decreased, presumably leading to the accumulation of 17β-estradiol in mammary tumor tissue [48]. Since estrogen sulfotransferase is an enzyme that inactivates 17β-estradiol, the levels of 17β-estradiol in mammary tumor tissue are increased [28,57]. Interestingly, melatonin reduces the activity of aromatase, sulfatase, and 17β-HSD1, and increases estrogen sulfotransferase expression, followed by declining estrogenic effects [48,57,58].

### 3.2. Melatonin Exerts Anti-Aromatase Roles via Regulating Cyclooxygenase (COX) Gene Activity

**Figure 1:**
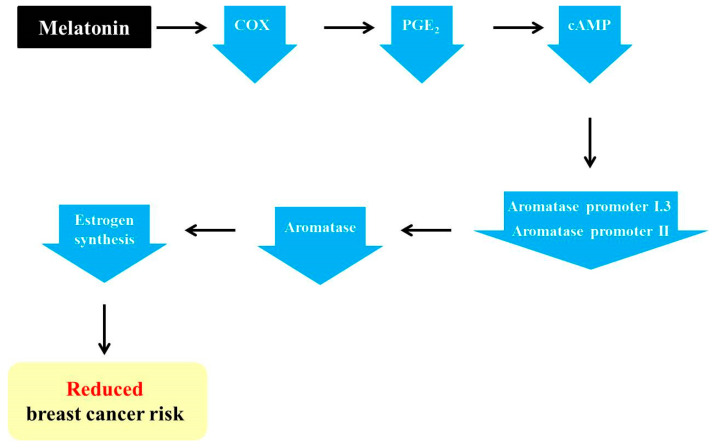
Melatonin-induced reduction in the risk of breast cancer via its regulation of aromatase promoters and related genes. Melatonin downregulates the levels of cyclooxygenase (COX) enzymes and leads to a reduction in prostaglandin E2 (PGE_2_). In turn, cAMP is also decreased, resulting in a decline in aromatase promoters I.3 and II and, ultimately, aromatase. Then, the decreased aromatase level impedes estrogen synthesis, thereby reducing the risk of breast cancer.

The pineal hormone melatonin is known to have oncostatic functions in hormone-dependent mammary tumors [59]. The regulatory functions of melatonin on the estrogen signaling pathway have made this hormone one of the potential tumor suppressive molecules [60]. Melatonin has been reported to act both as a SERM and a SEEM [61]. Moreover, this pineal hormone facilitates adipocyte differentiation and reduces aromatase activity by reducing estrogen production in the cells adjacent to tumor cells [49]. In addition, melatonin indirectly regulates estrogen synthesis by inhibiting aromatase activity. As described above, the activation of aromatase promoters II and I.3 causes increased aromatase expression in cancerous breast tissue [27]. Furthermore, increases in aromatase promoters II and I.3 are associated with increased cAMP levels via cancerous prostaglandin E2 (PGE_2_)-secreting epithelial cells [49,62]. It has been discovered that melatonin downregulates the gene expression of aromatase promoters II and I.3 [44,63,64]. Melatonin is known to downregulate the activation of the upstream COX2 pathways, including ERK1/2, JNK, p38 MAPK, and NF–NF-κB [65]. Melatonin-mediated downregulation of COX enzymes reduces PGE_2_, which is a tumor promoter generated by COX2 and is responsible for cell proliferation, death, and angiogenesis [66,67]. Then, diminished PGE_2_ reduces intracellular cAMP, leading to the decreased activation of promoters I.3 and II, as well as decreased aromatase levels. In turn, estrogen levels are also decreased due to the reduction in aromatase, ultimately followed by the suppression of breast cancer progression (Figure 1) [27,67].

### 3.3. Melatonin Increases the Efficiency of Conventional SEEMs and SERMs

**Figure 2:**
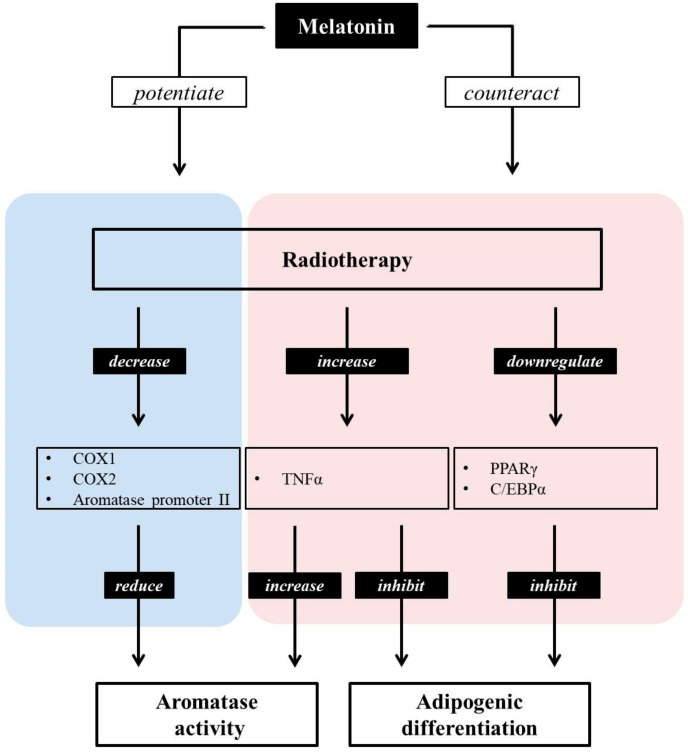
Melatonin can both potentiate and counteract the effects of radiotherapy. Radiation induces a decrease in adipocyte differentiation via downregulating PPARγ and C/EBPα, as well as by increasing TNFα. Furthermore, it reduces aromatase activity by decreasing COX1/2 and aromatase promoter expression. The pineal hormone melatonin potentiates the inhibitory role of aromatase activity, counteracting its suppressive role in the adipocyte differentiation of radiotherapy. Moreover, the increase in aromatase activity following enhanced TNFα levels due to radiotherapy may be counteracted by melatonin.

As described above, aromatase transforms testosterone and androstenedione into estradiol and estrone [41]. Among these hormones, androstenedione and estrone are low-activity steroids, while testosterone and estradiol possess a high level of activity [68,69]. Low-activity steroids (androstenedione and estrone) are converted into high-activity steroids (testosterone and estradiol) through catalyzation by 17β-hydroxysteroid dehydrogenase type 1, while 17β-hydroxysteroid dehydrogenase type 2 catalyzes the transformation of testosterone and estradiol into androstenedione and estrone, respectively [68,69]. In breast cancer, the production of these steroids is biased toward the creation of more active steroids [70]. In this way, the production of steroids with high activity is suppressed by melatonin. This hormone inhibits the expression and activity of 17β-hydroxysteroid dehydrogenase type 1, which controls the production of active estrogens from low-activity steroids [71]. Melatonin acts as a SEEM by suppressing the expression of enzymes in charge of producing more active steroids from those with a low biological activity, including aromatase, 17β-HSD1, and estrogen sulfatase [71,72]. In other words, melatonin may act as a SEEM, suppressing the biased production of active estrogens. In addition to the role of melatonin as a SEEM, this hormone may also be able to act as a SERM. Melatonin suppresses the binding of estrogen to Erα, meaning that melatonin regulates the proliferation, invasion, protein levels, growth factor expression, and expression of proto-oncogenes, including hTERT, p53, p21, TGFβ, and E-cadherin, of estrogen-dependent breast cancer cells [72]. These SERM actions of melatonin are known to be exerted in an MT1 melatonin receptor-dependent manner, rather than in an ERα-dependent manner. When melatonin binds to the MT1 receptor, this melatonin–MT1 complex reduces ligand–receptor transactivation, interrupting estrogen–ERα binding [73,74,75]. Interestingly, exposure to estrogen in rat ovaries has been shown to cause the downregulation of MT1 melatonin receptors [76]. As well as working as a SERM and SEEM, melatonin also enhances the effects of conventional SEEMs and SERMs. The antiestrogenic effect of tamoxifen has been found to be augmented by the physiological concentration of melatonin, suggesting that this hormone could act as a sensitizing molecule to conventional SEEM [77]. Similarly, the non-steroidal aromatase inhibitor aminoglutethimide shows increased efficiency when cells are pretreated with melatonin, as evidenced by the reduced aromatase mRNA expression in MCF7 cells following pre-exposure to 1 nM of melatonin [78]. Moreover, melatonin seems to be able to alleviate the side effects of aromatase inhibitors. It has been found that the hepatic function disturbances and hepatic toxicity caused by the aromatase inhibitor letrozole are improved by subcutaneously injected melatonin (0.5 mg/kg/day) in female rats [79]. In mammary tumors, adipocytes are found adjacent to tumor cells, and they may engage in cancer development with the potential of triggering cancer cell proliferation, migration, invasion, and resistance [80,81]. It has been documented that irradiation exposure inhibits adipocyte differentiation by downregulating the expression of two major adipogenic regulators, i.e., peroxisome proliferator-activated receptors (PPARγ) and cytidine-cytidine-adenosine-adenosin-thymidine (CCAAT)/enhancer-binding proteins α (C/EBPα) [49,82]. Furthermore, radiation has been found to elevate the expression of TNFα, which is known to facilitate breast cancer cell duplication through NF-kB-dependent pathways [49,83,84]. As described in Section 2.2, cytokines such as TNFα secreted from malignant cells hamper the normal differentiation of mammary adipose cells and increase aromatase activity. Radiation is able to reduce the mRNA levels of aromatase promoter II and COX1/2 expression, leading to decreased aromatase activity [49]. When co-treated with radiation, melatonin counteracts radiation-triggered adipogenic inhibition by increasing PPARγ and C/EBPα and by decreasing TNFα expression [49]. Moreover, this hormone enhances the radiation-induced reduction in aromatase promoter II, COX1, and COX2 expression. In this regard, the hormone melatonin is considered to counteract the side effects, and also to encourage the positive functions provided by radiation by promoting adipocyte differentiation and by repressing aromatase activation (Figure 2).

### 3.4. Melatonin Potentiates the Anti-Aromatase Effect of Radiation and Its Presumable Link to p53

Radiotherapy is one of the most widely used therapeutic choices for cancer, though it accompanies numerous unavoidable side effects in spite of recent technological advancements. Therefore, researchers have concentrated on how to reduce the harmful side effects on normal tissues. Several studies have insisted that melatonin is able to enhance the therapeutic effect of radiation and simultaneously protect non-cancerous cells from side effects following radiotherapy [28]. Indeed, melatonin has been proven to potentiate the inhibitory effect of radiation on aromatase expression and activity. The irradiation of 8 Gy combined with pretreatment with a physiological concentration of melatonin (1 nM) leads to a comparatively drastic reduction in aromatase activity and mRNA expression; radiation alone causes 40% and 50% reductions, whereas pretreatment with melatonin before radiotherapy leads to 70% and 75% reductions in aromatase activity and mRNA expression, respectively, leading to the sensitization of breast cancer cells to radiotherapy [85]. The tumor suppressor p53, which is known to play a role in apoptosis, DNA repair, and cell cycle arrest has been reported to be a negative modulator of aromatase in breast cancer [86,87,88]. As melatonin treatment prior to radiotherapy sensitizes breast cancer cells to radiation by downregulating DNA repair and promoting cell cycle arrest, p53 upregulation and consequent aromatase downregulation might represent the link between melatonin and its radiation-sensitizing effect on breast cancer cells.

### 3.5. Melatonin Enhances Anti-Angiogenic Function and Suppresses the Disadvantages of Chemotherapeutic Agents

It has been widely demonstrated that angiogenesis is crucial in cancer treatment, as tumor progression is closely associated with increased angiogenesis. Angiogenesis results from the interplay between breast cancer, endothelial cells, and sex hormones [89]. The extensive formation of new blood vessels represents the vigorous growth of a tumor [90]. The angiogenesis-stimulatory property of estrogens in breast cancer has been well documented. The influence of estradiol on angiogenesis is mediated via its regulation of angiogenic ligands, including vascular endothelial growth factor (VEGF), the soluble form of the VEGF receptor-1 (sVEGF-1), and VEGF receptor-2 (VEGF-2). Concretely, estradiol is able to stimulate VEGF and VEGF-2 and inhibits VEGFR-1, which is a negative regulator of VEGF-mediated angiogenesis, indicating the angiogenesis-favorable characteristics exerted by the hormone [89]. Melatonin appears to have an inhibitory role in angiogenesis under pathological conditions, including cancer [91]. Its neovascularization-suppressive properties have been suggested to be derived from the downregulation of hypoxia-inducible factor-1α (HIF-1α) and its downstream gene, vascular endothelial growth factor (VEGF) [92]. VEGF has been suggested to be a key factor of angiogenesis in cancer, owing to its neovasculatory function in human breast cancer [93]. Melatonin-induced prevention of HIF-1α nuclear translocation and the subsequent decrease in VEGF expression may hinder the angiogenic gene complex consisting of HIF-1α, phosphor-STAT3, and CBP/p300 in cancer [94].

One chemotherapeutic agent, vinorelbine, disrupts capillary tubule area formation by approximately 75% in human umbilical vein endothelial cells (HUVECs) as compared with non-treated controls, and this effect of vinorelbine is intensified by 1 mM melatonin treatment, suggesting that this pineal molecule could be a potential anti-angiogenic agent [23]. Apart from its benefits, vinorelbine may increase aromatase activity. Intriguingly, melatonin has been found to counteract the stimulatory effect of vinorelbine on aromatase activity by downregulating aromatase promoter I.7, which is one of the major aromatase promoters provoking aromatase transcription in breast cancer [23]. In other words, melatonin supplements the shortcomings, as well as highlights the advantages of chemotherapeutic agents.

Vascular endothelial cells adjacent to tumoral cells are a presumable source of estrogens, as they express aromatase [95]. These endothelial cells express aromatase promoter I.7, which is an endothelial-specific aromatase promoter region in breast cancer [31]. This promoter region may correlate with the angiogenesis of cancerous breast tissue [31]. Excessive expression of aromatase resulting from aromatase promoter I.7 in vascular endothelial tissue adjacent to cancerous breast tissue causes the development of breast tumors via the following two mechanisms: (1) Peripheral estrogen levels may be increased by excessive aromatase activity, leading to direct tumoral growth. (2) An increased concentration of estrogen due to excessive aromatase activity may encourage angiogenesis [96]. Aromatase mRNA is upregulated in endothelial cells via aromatase promoter I.7 [97]. Through the facilitation of the estrogen biosynthesis of endothelial cells, angiogenesis may contribute to the growth of ERα-positive tumors [97], that is, excessive aromatase expression and the consequent enhancement of estrogen synthesis and extensive angiogenesis seem to imply rapid tumoral growth in breasts. Melatonin is reported to downregulate aromatase promoter I.7, the endothelial-specific aromatase promoter region, and also to exert antiangiogenic effects [31]. Through the melatonin-induced downregulation of VEGF expression in mammary tumor cells, VEGF levels in vascular endothelial cells are also reduced [98]. Then, diminished VEGF levels play a suppressive role in the estrogen-producing cells adjacent to tumor cells [31]. In this way, decreased VEGF levels are considered to be vital for angiogenesis reduction in cancer [31].

### 3.6. Overexpression of the MT1 Melatonin Receptor Has an Aromatase-Suppressive Role and Mediates Oncostatic Action of Melatonin in the MCF7 Human Breast Cancer Cell Line

Several in vivo and in vitro studies have shown the downregulatory role of melatonin in aromatase activity. In MCF7 cells, RT-PCR analysis has revealed that the steady-state level of aromatase mRNA is downregulated by melatonin [26]. Likewise, melatonin-triggered aromatase suppression, a resultant decrease in estrogen synthesis, and reduced tumor growth have been found in mammary tumor rat models [99]. These oncostatic functions of melatonin are known to be exerted through specific receptors [100]. Melatonin exhibits its actions by activating its two high-affinity receptors, MT1 and MT2, throughout the tissues of the body [101,102]. These two melatonin receptors are the most well-characterized melatonin targets, belonging to the G protein-coupled receptor (GPCR) superfamily [102]. As described in Section 3.3, the SERM role of melatonin is associated with the MT1 melatonin receptor, rather than ERα [73,74,75]. Therefore, it can be inferred that the MT1 melatonin receptor is closely related to aromatase activity. The MT1 melatonin receptor is reportedly expressed in the estrogen receptor-positive human breast cancer cell line MCF7, which is sensitive to melatonin-mediated antiproliferation and anti-aromatase effects [26,75]. This human breast cancer cell line expresses aromatase [103,104], and the MT1 melatonin receptor [29,105]. Melatonin exerts an inhibitory role on the growth of MCF7 cells. However, it has been demonstrated that this growth-suppressive property of melatonin is enhanced when the MT1 melatonin receptor is overexpressed. Indeed, MT1-transfected MCF7 cells show a significantly enhanced suppression of growth [75]. The activation of the MT1 receptor leads to a reduction in cAMP [106]. Then, cAMP-mediated stimulation of aromatase promoters II and I.3 is increased, since cAMP is involved in the regulation of these aromatase promoters in breast cancer cells [107,108,109]. In this respect, it is speculated that melatonin has the potential to weaken aromatase activity by reducing cAMP levels. The enhanced tumor growth-suppressive role of melatonin appears to be associated with a reduction in aromatase activity following MT1 melatonin receptor overexpression. In MT1-transfected MCF7 cells, a 50% reduction in aromatase activity has been found as compared with vector-transfected cells, indicating that the MT1 melatonin receptor may enhance the inhibitory role of melatonin in relation to aromatase activity and expression [100]. In the same manner, MT1-transfected MCF7 cells in a medium without estrogen show enhanced aromatase suppression as compared with vector-transfected cells in the same medium [31]. Considering that this medium is used to measure aromatase activity, it appears to be more sensitive to melatonin in MT1-overexpressed cells. Plus, implantation with MT1-overexpressed MCF7 cells in mice followed by melatonin administration leads to a 60% reduction in palpable tumors as compared with mice injected with vector-transfected cells [110]. In short, the MT1 melatonin receptor is critical in order for melatonin to exert its oncostatic functions in breast cancer, suggesting that the MT1 melatonin receptor could be a key mediator in breast cancer-related melatonin pathways.

## 4. Conclusions and Perspectives

The suppression of estrogen synthesis is fundamental for breast cancer therapy, due to the strong link between estrogen and breast cancer progression. To block estrogen synthesis, the inhibition of aromatase expression is one of the crucial strategies, as this enzyme catalyzes estrogen transformation. The pineal hormone melatonin may be suggested as an oncostatic molecule based on its regulatory roles in aromatase activity and expression. By modulating the expression of aromatase promoters and related enzymes, melatonin reduces aromatase activity and contributes to a decline in estrogen synthesis. Moreover, this hormone appears to enhance the anti-aromatase effect of radiation, and offsets the unwanted aromatase increases caused by chemotherapeutic agents. Accordingly, these pleiotropic effects of melatonin would make this molecule a possible therapeutic option for breast cancer treatment.

## Data Availability

Not applicable.
